# Selenium and thyroid diseases

**DOI:** 10.3389/fendo.2023.1133000

**Published:** 2023-03-24

**Authors:** Fei Wang, Chunyu Li, Shaoxin Li, Lili Cui, Junyu Zhao, Lin Liao

**Affiliations:** ^1^ Department of Endocrinology and Metabology, The First Affiliated Hospital of Shandong First Medical University & Shandong Provincial Qianfoshan Hospital, Jinan, China; ^2^ Department of Endocrinology and Metabology, The First Affiliated Hospital of Shandong First Medical University & Shandong Provincial Qianfoshan Hospital, Shandong Key Laboratory of Rheumatic Disease and Translational Medicine, Shandong Institute of Nephrology, Jinan, China; ^3^ Department of Endocrinology and Metabology, Shandong Provincial Qianfoshan Hospital, Cheeloo College of Medicine, Shandong University, Jinan, China

**Keywords:** selenium, selenoprotein, thyroid disease, oxidative stress, iodine

## Abstract

Selenium, a non-metallic element, is a micronutrient essential for the biosynthesis of selenoproteins containing selenocysteine. In adults, the thyroid contains the highest amount of selenium per gram of tissue. Most known selenoproteins, such as glutathione peroxidase, are expressed in the thyroid and are involved in thyroid hormone metabolism, redox state regulation, and maintenance of cellular homeostasis. Some clinical studies have shown that lack of selenium will increase the prevalence of several kinds of thyroid diseases. Selenium treatment in patients with Graves’ orbitopathy has been shown to delay disease progression and improve the quality of life. Selenium supplementation in Hashimoto’s thyroiditis was associated with the decreased levels of anti-thyroid peroxidase antibody and improved thyroid ultrasound structure. In thyroid cancer, various selenium supplements have shown variable anticancer activity. However, published results remain the conflicting and more clinical evidence is still needed to determine the clinical significance of selenium. This article reviews the strong association between selenium and thyroid disease and provides new ideas for the clinical management of selenium in thyroid disease.

## 1 Introduction

In 1817, the Swedish chemist Berzelius discovered a non-metallic element and named it selenium (Se). Se is an essential trace element for human body (1). In the 1980s, it was found that supplementation with sodium selenite could improve chondrodystrophy (Kashin-Beck disease) and juvenile cardiomyopathy (Keshan disease) which were caused by Se deficiency. That was the first time Se was found to be useful in clinical treatment. With the gradual increased understanding of Se, it has been proposed that there is a U-shaped curve between Se status and the health status of the organism (2). Patients with Se deficiency can benefit from Se supplementation, while Se supplementation in people with adequate Se levels can exacerbate the risk of certain diseases (3). The thyroid is one of the highest content of Se in the body organs, it is interesting to note that in the case of Se deficiency, the Se content of thyroid gland is also high (4). Se is present in selenoproteins in the form of selenocysteine, which is involved in constituting the active center of selenoproteins. It plays an important role in the metabolism of thyroid hormones and in the fight against oxidative stress (5). This highlights the uniqueness of human thyroid and the importance of Se to the thyroid gland. Although the relationship between Se and thyroid diseases is not well established and needs to be explored in depth. Low Se levels are currently considered to be one of the independent risk factors for thyroid diseases and Se supplement treatments for patients with low Se levels are thought to be generally beneficial for thyroid diseases.

## 2 Se is closely related to the metabolism of thyroid hormones

### 2.1 Se and selenoprotein

Se is absorbed by the body and involved in the synthesis of selenoproteins. It exerts biological functions such as antioxidant and metabolic regulation through selenoproteins, which are key biomolecules. To date, we have identified 25 genetically encoded selenoproteins in human, including glutathione peroxidase (GPx), thioredoxin reductase (TXNRD), and iodothyronine deiodinases (DIOs), which have a wide range of functions, from anti-inflammatory and antioxidant activities to thyroid hormone metabolism.

### 2.2 Selenoproteins are involved in the metabolism of thyroid hormones

**Figure 1:**
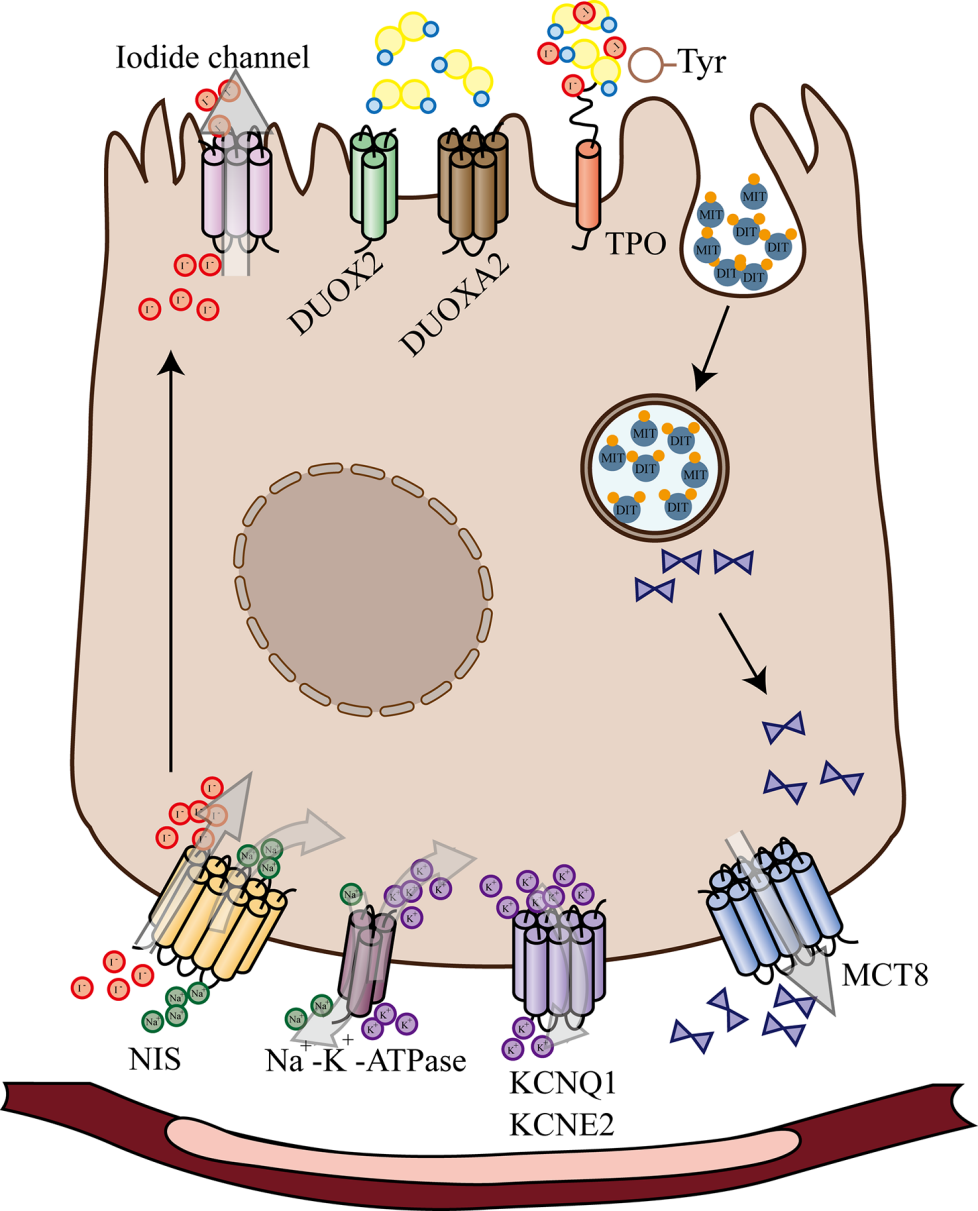
Schematic Diagram of Thyroid Hormone Biosynthesis and Release. After entering the thyroid cells, iodine ions are activated by H2O2 under the action of thyroid peroxidase. The activated iodine binds to tyrosine residues on thyroglobulin molecules under the action of TPO to produce monoiodotyrosine and diiodotyrosine. NIS, sodium/iodide symporter; KCNQ1 and KCNE2, Voltage-gated K^+^ channels; MCT8, SLC16A2 monocarboxylate transporter 8; DUOX2, dual oxidase 2; DUOXA2, maturation factor of dual oxidase 2; TPO, thyroid peroxidase; MIT, monoiodotyrosine; DIT, diiodotyrosine.

After entering the thyroid cells, iodine ions are activated by H2O2 under the action of thyroid peroxidase (TPO). The activated iodine binds to tyrosine residues on thyroglobulin molecules under the action of TPO to produce monoiodotyrosine (MIT) and diiodotyrosine (DIT), which are subsequently coupled to produce T3 or T4 (**Figure 1**). Activation and deactivation of thyroxine need the participation of DIOs to complete (6–8).

When iodine is sufficient in the body, the production of H2O2 is the step that limits the synthesis of thyroid hormones; when iodine is lacking, under the stimulation of high TSH, thyroid cells produce more H2O2, whose accumulation gradually damages thyroid cells. Selenoproteins such as GPXs and TRs can scavenge H2O2, protect cell membrane structure and function, repair the site of molecular damage, achieve anti-oxidative stress and local protective effects against oxidative stress or inflammation. In Se deficiency, GPx activity decreases, degradation of H2O2 is reduced, thyroid cells are less resistant to oxidative stress, apoptosis and cell death occur (9). On the other hand, the activity of DIOs is reduced in Se deficiency, thyroxine is not activated and affects the thyroid hormones to perform their biological functions.

## 3 Se deficiency is one of the risk factors for many thyroid diseases

### 3.1 Se and Graves’ disease

The main clinical manifestation of Graves’ disease (GD), also known as toxic diffuse goiter, is thyrotoxicosis caused by excessive production of thyroid hormones. In this hypermetabolic state, the body releases a large number of reactive oxygen species (ROS), which can lead to thyroid epithelial cell damage, autoantigen activation of the immune system, and induction of autoimmune deterioration. Graves’ orbitopathy (GO) is the most prominent and common extrathyroidal manifestation of GD, characterized by the production and accumulation of glycosaminoglycans (especially hyaluronic acid) in the retrobulbar and periorbital tissues causing protrusion of the eyeball and restriction of ocular muscle movement (10). A large number of clinical trials have demonstrated the efficacy of Se in the treatment of Graves’ hyperthyroidism, but the results have been somewhat contradictory. We conducted a screening of clinical controlled trials on GD using PubMed and Cochrane library databases with the search terms: “(Graves’ disease OR hyperthyroidism) AND Se”. After analysis of 11 clinical trials with full text that are eligible, 9 trials confirmed that Se supplementation resulted in faster achievement of normal thyroid function in patients with hyperthyroidism, but 2 still did not show an adjuvant effect of Se. The details are shown in **Table 1**.

**Table 1:** Characteristics of 11 included studies.

First author Year	Country	Characteristics of participants	Interventions dose	Number of participants, n		Mean age, year		Male (%)		Follow-up time	Outcome index	Outcome
				Se	control	Se	control	Se	control			
Bacic Vrca, V. 2004 (11)	Croatia	newly detected GD	capsule of antioxidants (include 60ug Se)	29	28	–	–	14	4	2 months	FT4, FT3, TSH, ferritin, transferrin glucose, uric acid and TAS	Experimental group reached normal thyroid function faster
Bacic Vrca, V. 2004 (11)	Croatia	newly detected GD	capsule of antioxidants (include 60ug Se)	29	28	–	–	14	4	2 months	FT4, FT3, TSH, serum selenium GPx activity	Experimental group reached normal thyroid function faster
Bacic Vrca, V. 2005 (12)	Croatia	newly detected GD	capsule of antioxidants (include 60ug Se)	27	28	44.0± 12.0	41.0± 14.0	15	4	2 months	FT4, FT3, TSH, SOD activity, Cu and Zn concentrations in erythrocyte lysate	Experimental group reached normal thyroid function faster
Marcocci, C. 2011 (13)	Holland	mild signs or symptoms of GO of less than 18 months’ duration	sodium selenite (100ug twice/day)	54	50	43.0± 11.0	44.6± 10.7	11	18	12 months	overall ophthalmic assessment and the GO-QOL score	Selenium treatment is associated with improved quality of life and reduced ocular involvement
Vrca, V. B. 2012 (14)	Croatia	newly detected GD	capsule of antioxidants (include 60ug Se)	27	28	44.0± 12.0	41.0± 14.0	15	4	2 months	FT4, FT3, TSH, triglyceride, LDL- and HDL-cholesterol	Experimental group reached normal thyroid function faster
Calissendorff, J. 2015 (15)	Sweden	newly diagnosed and untreated GD(aged 18–55)	Se yeast(200ug/day)	19	19	35.0	44.0	21	16	36weeks	FT4, FT3, TSH, TRAb, TPOAb and self-rated symptoms	Selenium supplementation promotes biochemical recovery
Kahaly, G. J. 2017 (16)	Germany	untreated GD patients	sodium selenite (300ug/day)	35	35	44.5 (13.8)	44.5 (13.4)	20	26	36weeks	the response rate at week 24 and the remission/recurrence rate at week 36	Selenium supplementation did not affect the response or recurrence rate of GD
Leo, M. 2017 (17)	Italy	newly diagnosed hyperthyroid GD	L-selenomethionine (166ug/day)	15	15	43.0± 11.0	38.0± 11.0	7	13	90days	control of hyperthyroidism, clinical and biochemical manifestations of it	No auxiliary role of selenium in GD was shown
Xu, B. 2019 (18)	China	newly diagnosed hyperthyroid GD	selenium tablets (150ug twice/day)	44	50	38.89±11.59	40.20±12.63	32	38	6months	FT4, FT3, TSH, TRAb, TPOAb, TGAb	Combined use of selenium improves thyroid viability in patients
Almanza-Monterrubio, M. 2021 (19)	Mexico	mild and active GO by CAS > 3(≥18years old)	selenium tablets (100ug twice/day)	15	15	40.7± 10.5	42.5± 11.8	20	27	6months	visual acuity in LogMAR scale, palpebral aperture (mm), proptosis measured with Hertel exophthalmometer and CAS	Oral Selenium Improves Disease Activity in Patients with mild GO
Gallo, D. 2022 (20)	Italy	newly diagnosed GD with serum Se <120 mcg/l and plasma VitD <30 ng/ml	selenomethionine 83 mcg + selenium yeast 17 mcg(180 days stop)	21	21	45.8± 9.3	47.7± 11.4	19	5	270days	FT4 levels mean decrease from baseline to 180 days.biochemical marker, clinical parameters and QoL scores at 45, 180 and 270 days.	Achieving optimal Se and VitD levels can improve the early efficacy of MMI treatment

GD, Graves' disease; GO, Graves' orbitopathy; CAS, clinical activity score; Se, selenium; FT4, free thyroxine; FT3, free triiodothyronine; TSH, thyroid stimulating hormone; TAS, total antioxidant status; GPx, glutathione peroxidase; SOD, superoxide dismutase; TRAb, thyrotropin receptor antibody; TPOAb, thyroid peroxidase antibody; TGAb, thyroglobulin antibody; GO-QOL, Graves' orbitopathy–specific quality-of-life questionnaire.

#### 3.1.1 The role of Se in GD

A large number of studies have now confirmed Se deficiency as a risk factor for GD in areas with adequate soil Se levels (21–26). Notably, a cross-sectional study conducted in an area with poor soil Se showed that Se levels in patients in GD with or without GO were lower than in normal healthy controls (27), which would seem to be able to suggest that relatively low Se level is an independent risk factor for GD.

Based on the damage to thyroid cells caused by low Se level, researchers have raised the possibility that Se supplementation may be beneficial in GD and have conducted a series of clinical studies (16–18, 20, 28–30). In these studies, patients in the experimental group were often treated with Se supplements or antioxidants containing Se in combination with antithyroid drugs (generally methimazole, MMI). Interestingly, these results were not always consistent. During the study by Nordio, M., subjects took one tablet containing 500 mg of L-carnitine and 83ug of Se (L-Carn + Se) orally daily for 1 month. It showed significant relief of symptoms associated with subclinical hyperthyroidism and improved the quality of life of the patients, but no significant effect was seen in terms of thyroid function (28). In contrast, the results of Gallo, D. showed that MMI + antioxidants (83 ug selenomethionine + 17 ug Se yeast + vitamin D) treatment for 6 months improved thyroid activity more effectively than MMI use that alone (20). We speculate that this difference in results might be related to the type of Se supplementation, dose, duration of treatment and the status of other nutrients in the subject’s organism. In studies which only additional Se was added to experimental groups, the findings were also variable. The results of Leo, M. showed that serum Se levels and selenoprotein concentrations were not associated with shortterm control of GD (17), while a randomized controlled trial by Xu, B. demonstrated that Se supplementation improved thyroid activity (18). The former study was conducted in an area with sufficient Se and therefore failed to show a short-term therapeutic effect of Se in hyperthyroidism. This difference in study premise may explain the difference in results.

Although the current findings are ambiguous and evidence from clinical trials does not favor the use of Se as a routine treatment option in GD, nor does the use of Se supplementation affect the remission and recurrence rates of GD (16). However, it is undeniable that correction of moderate to severe Se deficiency has a positive impact on the prophylaxis of GD.

#### 3.1.2 The role of Se in GO

Current studies suggest that fibroblasts are distributed in the posterior globular connective tissue and ocular myofilament. They are target and effector cells of GO autoimmune response. In a study by Rotondo Dottore, G., it was mentioned that H2O2 has a dual effect on fibroblast cell proliferation, with low concentrations of H2O2 inducing proliferation and releasing cytokines, while high concentrations of H2O2 are cytotoxic, when cell viability decreases (9, 31). By further treating adipose/connective tissue in the orbital region with selenocysteine (SeMCys), Rotondo Dottore, G. and his team found that SeMCys appeared to reduce the toxic effects of H2O2 by reducing cell necrosis and apoptosis. In another study, Kim, B. Y. confirmed the beneficial effects of Se on orbital fibroblasts by primary culturing of orbital specimens from GO patients and healthy subjects with selenite (32). These findings seem to be interesting and they seem to indicate that Se also has a dual role in orbital fibroblasts, i.e., under conditions of oxidative stress without cytotoxicity, Se can inhibit the release of pro-inflammatory factors and hyaluronic acid; under conditions of cytotoxic oxidative stress, Se can prevent cellular damage and the release or exposure of autoantigens as well as reduce the toxic effects of reactive oxygen radicals. Excitingly, in addition to improving the antioxidant capacity of the body, Se is also thought to directly affect the sympathetic tone of opercular muscles and reduce inflammation in the muscles of the eyelids (33).

The effect of Se on patients with mild GO was further confirmed by a randomized clinical controlled trial conducted by Marcocci, C. et al. (13). In this study, subjects were arbitrarily assigned to one of the sodium selenite (100 micrograms twice daily), pentoxifylline (600 mg twice daily), or placebo (twice daily) therapy groups for a duration of 6 months. It was then followed up for 6 months after treatment was stopped. At the end of treatment, the investigators found that more patients in the Se group had improved the quality of life, while significantly fewer patients had disease progression. Encouragingly, Se also expressed a sustained beneficial effect on GO during the follow-up period. Notably, this study was conducted in different regions of Europe with different Se levels, so it is unclear whether the effectiveness of Se and the generalizability of the result were confounded by other confounding factors such as the effect of baseline Se levels.

Although the actual efficacy of Se in GO is uncertain, it is generally considered to be beneficial in the treatment of mildly active GO (19) and has been used in clinical practice (17, 34, 35). It is instructive that the 2021 EUGOGO guidelines include Se supplementation in the treatment regimen for mild GO, recommending a 6-month treatment with Se preparations for patients with mild GO of short duration to prevent the progression to more severe forms of GO (34).

### 3.2 Se and Hashimoto’s thyroiditis

In recent years, the incidence of Hashimoto’s thyroiditis (HT) has been increasing with the wide application of thyroid ultrasound, fine needle puncture biopsy and other testing techniques. However, due to the strong occult nature of the disease and atypical clinical symptoms, a large number of HT have not been diagnosed. Its epidemiological details are still very limited. HT is known to be the leading cause of primary hypothyroidism in areas where iodine is abundant. The prevalence of hypothyroidism varies from 0.2% to 5.3% in different regions. This proportion varies with geography, genetic factors, gender and age. The prevalence of overt hypothyroidism in the general population ranges from 0.2% to 5.3% in Europe and only 0.3% to 3.7% in the United States. A meta-analysis based on the human genome showed that high-risk individuals were 2.5 times more likely to have hypothyroidism than those at low genetic risk. Stratified analysis of gender shows that women are 10 times more likely to suffer from hypothyroidism than men. The prevalence increased to more than 20% for women in the higher age group (≥75 years) (36, 37). Due to the increased prevalence of HT (38–40), numerous studies have been conducted by researchers to address its etiology and treatment.HT occurs when the immune system produces autoantibodies that attack the thyroid gland and some thyroid follicular cells are destroyed, resulting in insufficient thyroid hormone secretion and compensatory proliferation of undamaged thyroid follicular cells to produce more thyroid in order to maintain the normal function of organism hormones. The pathological manifestations of HT are often lymphocyte infiltration, follicular cell atrophy and glandular fibrosis. A 2021 review of autoimmune thyroiditis (AITD) clearly identified Se as an important risk factor for HT (41). Some cross-sectional studies have also confirmed low Se levels in HT patients (27, 42). Some researchers have detected a general deficiency of the antioxidant Se in HT patients with subclinical hypothyroidism in the area of Ankara, Turkey, which is iodine-rich. In the case of the cross-sectional study conducted in a Se-deficient area, although the difference between HT and controls was not statistically significant, Se levels of HT patients were lower than those of controls, and we speculate that the results may be limited by the size of the study with too few sub-jects. In conclusion, at this stage of the study, we still consider Se deficiency as a risk factor for HT. This seems to be related to the reduced activity of Se-dependent enzymes such as GPx, which has strong antioxidant activity to scavenge excess superoxide in the thyroid and maintain the integrity of cell membranes.

Se deficiency is often accompanied by a loss of immune function (43, 44). In cellular immunity, Se may reduce thyroid antibodies by upregulating activated Treg cells (45). Se deficiency may upregulate Th1/Th2 effectors and enhances immune responses. The possible therapeutic effect of Se in HT to improve immune function was validated in a prospective study conducted in 2022, which showed that Se supplementation with 100ug per day improved thyroid function and the quality of life of patients by decreasing interferon gamma concentrations and increasing interleukin 1β concentrations (46).

In addition, back in 2017, researchers studied the immunological effects of selenomethionine (SeMet) in 21 patients who had normal thyroid function with HT (47). The patients were treated with myoinositol plus Se (600mg/83ug) tablets twice daily for 6 months. Excitingly, in addition to the significant decrease in TSH levels, there was also a lever reduction in serum CXCchemokineligand-10 (CXCL10) chemokine which were induced by IFN-γ. CXCL10 is released by thyroid cells in response to IFN-γ stimulation. Its serum level is often proportional to the percentage of lymphomonocyte infiltration in thyroid tissue and the degree of thyroid destruction (48, 49). The immunoregulatory effect of myoinositol combined with Se on CXCL10 indicated that it could reduce the immune response of the body (50). However, the specific mechanism of this process remains unclear, and the specific role of the antioxidant Se in this process needs further research to clarify.

The autoimmune process of HT has a specific elevation of thyroid peroxidase antibodies (TPO-Ab) in addition to the chronic lymphocyte invasion of the gland. This specific elevation suggests that disorders of our humoral immunity may be one of the risk factors associated with HT. Based on characteristic serological markers of HT, a prospective clinical trial demonstrated that Se supplementation reduced TPO-Ab titers and improved the quality of life of patients (45), which is consistent with the results of several intervention studies or meta-analyses (51–55). It is worth exploring that, the high antibody group (TPO-Ab>200) also had a decrease in thyroglobulin antibody (TG-Ab) at 6 months, which seems to indicate that the high antibody group could benefit more significantly from treatment with Se supplementation. Karanikas did not observe inhibition of TPO-Ab titers by Se in their patients (56). The reason for this is unclear, but the result of this study does not deny the therapeutic effect of Se on HT.

Notably, women during pregnancy and delivery are a special population of HT patients. Pregnant women with TPO-Ab-positive have a higher risk of preterm delivery and miscarriage, as well as the development of postpartum thyroid dysfunction (PPTD) and eventually permanent hypothyroidism (57). A prospective controlled study showed that supplementation with 200ug SeMet per day during pregnancy and postpartum, reduced the incidence of PPTD and hypothyroidism (58).

In HT patients with severe hypothyroidism, levothyroxine (L-T4) is the usual treatment strategy. In an open controlled trial of 60 patients with chronic lymphocytic thyroiditis (59), the combination of L-T4+ Se possessed better efficacy than L-T4 monotherapy compared to the L-T4 group, TPO-Ab and TG-Ab were significantly reduced. However, in another intervention trial with combination therapy (53), the risk of adverse effects was significantly higher in the Se supplementation group, which we speculate may be related to unclear baseline Se levels in the subjects and a narrower safety window during Se treatment.

In summary, Se deficiency appears to be a risk factor for HT and the current evidence does not justify the use of Se supplementation as part of the treatment of HT, despite its ability to improve immune function. If supplementation is indeed required, it must be preceded by a careful consideration of the patient’s Se baseline status, gender, weight and so on (60).

### 3.3 Dual role of Se deficiency in cretinism

**Figure 2:**
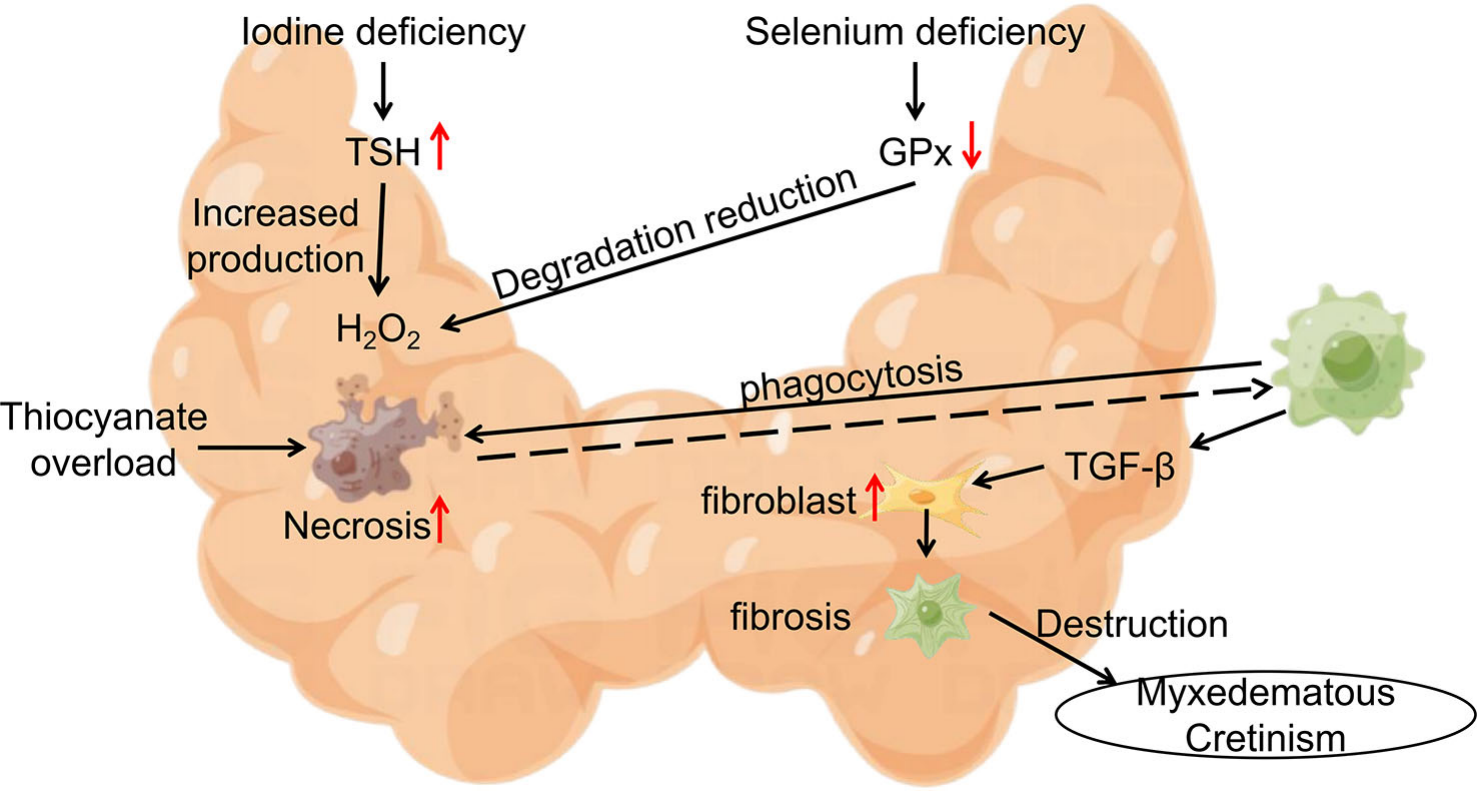
Epidemiological Model of Myxedematous Cretinism. Iodine deficiency, Se deficiency and thiocyanate overload are involved in the pathogenesis of myxedematous cretinism. TSH, Thyroid Stimulating Hormone; GPx, glutathione peroxidase; TGF-β, transforming growth factor-β.

Cretinism often occurs in areas where endemic goiter is prevalent, with mucinous edema cretinism being the main manifestation of hypothyroidism without goiter. Researchers’ intervention studies on cretinism originated from the emergence of familial aggregates of cretinism in Central Africa (northern Zaire). Contempré, B. and his team constructed a model of the epidemiology of mucinous edema cretinism (61) (**Figure 2**). In this model, iodine deficiency and Se deficiency exacerbate H2O2 accumulation, Se deficiency decreases cellular defense, promotes thyroid cell fibrosis, increases the inflammatory response following thyroid cell necrosis, and thiocyanate overload triggers follicular cell necrosis. The interaction and mutual promotion of these three factors are thought to be responsible for the concentrated epidemic of cretinism in Central Africa (62–66).

It is thought-provoking that Se supplementation in iodine-deficient populations in this region can have serious and incalculable consequences (64, 67). In 1988, Contempre, B. and his team treated 26 children with cretinism living in northern Zaire with 50ug of Se per day for 2 months, but the results of the study showed a further decrease in the already impaired thyroid function of the subjects. This result may be due to the low percentage of functional thyroid tissue in children with cretinism in which T4 is converted to active T3 at the periphery in the presence of Se supplementation only. At this time, the body’s demand for thyroid hormones increases, and the small amount of iodine stored in the gland is consumed more quickly without being replaced, resulting in a more rapid loss of thyroid status. In contrast, in patients with congenital hypothyroidism in areas without iodine deficiency treated with 20-60 ug selenomethionine for 3 months (68), Se supplementation was found to reduce serum thyroglobulin. In addition, Se supplementation improved hypothalamic-pituitary feedback to thyroid hormones, and thyroid stimulating hormone (TSH) returned to within the normal range, thereby reducing the stimulatory effect of TSH on thyroid tissue. The comparison of the results of these two studies seems to indicate that Se deficiency protects to some extent the thyroid function in patients with cretinism in Central Africa.

This enlightens us that Se supplementation should not be done indiscriminately in patients with congenital hypothyroidism (CH). For cretinism in iodine-deficient areas, Se supplementation should not be promoted prior to iodine supplementation or when there is no plan for iodine supplementation, as stated by the American Thyroid Association, “The differential intakes of iodine, Se, or both in different regions must be considered before any action for Se supplementation is taken” (69).

### 3.4 Se and thyroid tumors

The incidence of thyroid cancer has been on the rise in the past decades, increasing faster than any other cancer (70, 71). In addition to unchangeable external conditions such as age, gender, race and genetic susceptibility to thyroid cancer (72–74), the role played by the trace element Se in thyroid cancer has attracted the attention of researchers. Although most findings suggest a positive association between low Se level and the risk of thyroid cancer (24, 75–77), a prospective study conducted by Xu, X. and his team did not seem to find this association (78). From 1993-1998 researchers recruited 147,348 postmenopausal women through the Women’s Health Initiative (WHI) database and investigated the subjects’ dietary habits (Se intake) through a dietary questionnaire, and over the following 16 years, 442 cases of thyroid cancer were identified among the subjects. After adjusting for multiple covariates in the study, the researchers found no significant association between Se intake and thyroid cancer. This result is not consistent with previous studies that have investigated the link between low Se levels and thyroid cancer. In this regard, we speculate that the irrelevance of this study may stem from the method of Se measurement, which is available in whole blood, serum (79, 80), urine (81), nails (82), and questionnaires (83). The Se levels of the subjects in this study were inferred from the diet, so it has an information bias. Secondly, it was found that dietary Se intake was weakly correlated with peripheral Se levels such as whole blood and serum (84), and the intake of Se did not accurately reflect the relationship of serum Se and thyroid carcinoma, which may be another reason for the inconsistent results of this experiment. Finally, Xu, X.’s study was conducted in postmenopausal women whose Se intake was greater than or equal to baseline levels, and the subject’s condition was another major limitation of this trial. In conclusion, we suggest that Se deficiency promotes the risk of thyroid cancer, but more conclusive research evidence is still needed to conclusively determine this relationship (75).

In addition to increasing the risk of disease, Se deficiency is also strongly associated with the progression of thyroid cancer. Some findings showed that serum Se concentration was inversely correlated with disease stage (P=0.011), and low levels of serum Se were potentially associated with high stage of thyroid cancer (80). Although 25 selenoproteins are tissue-specific finetuned in Se deficiency (85) and deiodinase is a highly conserved selenoprotein, the content activity of deiodinase also decreases in severe Se deficiency, when the body synthesizes significantly less T3 and the inhibitory effect on TSH is diminished. Increased TSH fosters cAMP synthesis, which in turn initiates cAMP-dependent protein kinase signaling systems, to potentiate EGF-mediated cell proliferation, stimulating thyroid cell growth and promoting tumor cell proliferation, invasion and metastasis (86–88).

At the molecular level, researchers conducted a multiomics data mining study and found that multiple selenoproteins are lowly expressed in thyroid cancer (89), which is associated with reduced selenoproteins content and activity due to Se deficiency and is consistent with the results of several previous studies (90, 91).GPx3 is known to be the only member of the GPx family that can be secreted into the plasma and therefore it can play an important role in extracellular oxidative stress (92), which enhances the antioxidant defense of cells *via* a blockage of redox DNA destruction, thus reducing as well a reduction in the abundance of damaged cells (93). On the other hand GPx3 is negatively correlated with MAPK oncogenic signaling pathway (94), which suggests a potential antitumor effect in thyroid cancer, while a decrease in GPx3 levels predisposes to an increase in the size of primary tumors and the number of metastatic lymph nodes (95, 96).

At the cellular level, Erdamar, H. examined 41 tissue samples (including 9 papillary thyroid cancer tissues) (97) and found that their Se levels and GPx activity were lower than those of non-cancerous tissues, while malondialdehyde (MDA) concentrations were increased. This may be due to the vicious cycle of increased lipid peroxidation and free radicals in cancer cells, while the decrease in GPx activity makes cancer tissues more susceptible to the damaging effects of free radicals. In conclusion, Se can “boost” the immune and antioxidant capacity of the body and strengthen the immune defense.

In view of the role of Se deficiency in promoting thyroid tumorigenesis and progression, appropriate Se supplementation for differentiated thyroid cancer has been proposed to delay the disease progression and improve the prognosis (98).Kato, M. A. et al. treated thyroid cancer cells of different cell lines with selenomethionine (SeMet) (99) and found that SeMet could timedependently upregulate the expression of GADD family genes and arrest cells in cell cycle S phase or G2/M phase to inhibit the proliferation of thyroid cancer cells. This provides another great evidence for the clinical treatment of Se against cancer.

For differentiated thyroid cancer, surgical resection, radioiodine therapy, and TSH suppression therapy are often the three steps of its treatment. Radiation inflammation is the most common long-term complication during iodine therapy, often manifesting as dry mouth, altered taste, and dental caries (100, 101). Excitingly, Se supplementation may protect patients’ salivary glands from radiation (102). On the other hand, the use of antioxidants containing Se may reduce the oxidative stress state of the body during iodine treatment (103).

Although there are no recommendations for the addition of compounds containing Se to the medical therapy of thyroid cancer, their anticancer properties have attracted attention. It is worth noting that there is still a need for careful consideration as to whether to use Se-containing compound to intervene in the development and progression of cancer. Apart from possible selenosis caused by excessive Se accumulation, intervention with micronutrient Se in subjects with adequate Se levels failed to reduce the incidence of thyroid cancer (82).

## 4 Se supplementation and precautions

The daily intake of Se in human is determined by a combination of the Se value in dietary content, the intake of food and the configuration of the diet. The Se content of food is strongly dependent on the Se content of the soil in which plants and animals grow (104–106). In China, about 51% of the regions are deficient in Se (107) Se. A study by Dinh, Q. T. and his team also found that 39-61% of the Chinese population had daily Se intakes below standards according to WHO/FAO recommendations. It is known that Se cannot be synthesized in the human body and daily supplementation is required to meet the body’s Se requirements. Therefore, the need for Se supplementation to reduce disease incidence and delay disease progression in people with low baseline Se status has attracted the interest of researchers.

### 4.1 Types of Se supplements

Se occurs by two modes naturally: both inorganic Se and phytoactive Se. Inorganic Se has a greater cumulative toxicity and is not readily absorbed (108), and is not suitable for use in human. phytoactive Se generally exists as selenomethionine, which is biotransformed to synthesize specific selenoproteins and is not directly toxic even at a high doses (109). Ingested selenomethionine can bind to tissue proteins, especially muscle proteins, forming a reservoir that is slowly released according to the protein turnover rate in the body (42).SeSeSeSe

### 4.2 Factors affecting the absorption of Se supplements

- Vitamin E: Adequate vitamin E improves the body’s ability to utilize Se and can multiply the Se accumulation in the liver (110).
- Iron: Patients with iron deficiency anemia are often accompanied by a decrease in glutathione peroxidase activity. In this case, iron should be supplemented along with Se in order to restore its activity to normal levels in a timely manner (111–113).
- Thiamine-containing amino acids: Thiamine-containing amino acids are the raw materials for the synthesis of glutathione. It directly affects the synthesis of glutathione when the dietary content of thiamin-containing amino acids is low. Therefore, vegetarians with low protein intake have lower levels of both amino acids and Se in their bodies.
- Intestinal bacteria: Thyroid disease and intestinal disease commonly coexist, probably because of disruption of the intestinal barrier, where it is easier for antigens to pass through and react with the immune system or with extra-intestinal tissues to intersect and destroy thyroid cells. Lactobacillus and Bifidobacterium are positively associated with Se absorption, and these bacteria are reduced in autoimmune thyroid disease. Escherichia coli, Streptococcus faecalis, Clostridium difficile and certain Salmonella species can bind Se from the body to their own enzymes to use amino acids instead of thiamine-containing amino acids, and can also convert the soluble form of Se to an insoluble form, making it unavailable for absorption by the body (114).

### 4.3 Manifestations of Se toxicity

A study conducted in China showed that the lowest level of harmful effects observed for Se toxicity was 900-1000ug/day (115). Excessive Se intake can lead to adverse health problems and Se toxicity. The clinical features of Se toxicity mainly include brittle hair and nail loss, gastrointestinal disorders, rash, rickets and neurological disorders (116).

Based on the facts described above, Se status at baseline is the determining element for the assessment of Se supplementation needs. Serum Se measurements should be performed in patients before, during and after Se supplementation to assess the organism’s Se status.

### 4.4 Assessment of Se status

Se exists in the body in many forms and can be excreted in urine, feces and lungs. Common methods for determining Se include urine (81), blood (79, 80) and hair (117). About 50-60% of ingested Se is known to be excreted in urine (2, 118). By measuring it we can estimate Se intake over a short period of time.

Se levels in the blood often represent the state in which the body uses and accumulates. It is important to note that serum Se (S-Se) does not directly reflect the concentration of Se in tissues, and even normal S-Se content does not exclude the possibility of Se deficiency in the thyroid (119, 120). Selenoprotein P (Se-P) may be a better biomarker of Se nutritional status than S-Se. As the main transportation and storage protein of Se, low levels of Se-P will also ensure Se concentration in various tissues in the event of Se deficiency and disease (121). In the case of Se deficiency, Se-P will decrease first. After Se supplementation, the body will firstly increase the level of selenoproteins such as GPx and then Se-P to normal levels (122).

The Se in hair often represents the nutritional status of Se in the body over a period of weeks or even months. A study conducted in China showed that the Se content of hair is <0.20 mg/kg for Se deficiency, 0.20-0.25 mg/kg for marginal Se deficiency, 0.25-0.50 mg/kg for medium Se nutrition level, and ≥0.50 mg/kg for high Se nutrition level (117). However, whether the universality of this criterion is limited by ethnicity, region and gender remains to be further studied.

## 5 Summary

The microelement Se performs an essential role in maintaining normal body functions as well as the function of the thyroid axis. Se deficiency is a risk factor for many thyroid disorders, and Se supplementation offers new ideas for clinical practice in thyroid disorders. Although current recommendations for Se therapy extend only to GO patients, Se supplementation has been widely used by clinicians for a variety of other thyroid disorders. More reliable clinical evidence is still needed to determine the role of Se supplementation in thyroid disorders.

## Author contributions

FW: Literature retrieval, Paper writing and Paper submission. CL: Literature retrieval. SL: Literature retrieval. LC: Literature retrieval. JZ and LL: Article guidance, Paper revision and Paper submission. All authors contributed to the article and approved the submitted version.
